# Rice Seed Priming with Picomolar Rutin Enhances Rhizospheric *Bacillus subtilis* CIM Colonization and Plant Growth

**DOI:** 10.1371/journal.pone.0146013

**Published:** 2016-01-07

**Authors:** Akanksha Singh, Rupali Gupta, Rakesh Pandey

**Affiliations:** Department of Microbial Technology and Nematology, CSIR- Central Institute of Medicinal and Aromatic Plants, P.O. CIMAP, Lucknow 226015, India; Louisiana State University Agricultural Center, UNITED STATES

## Abstract

The effect of rutin, a bioflavonoid on the growth and biofilm formation of *Bacillus subtilis* strain CIM was investigated. In addition to swimming, swarming, and twitching potentials of *B*. *subtilis* CIM (BS), one picomolar (1 pM) of rutin was also observed to boost the biofilm forming ability of the bacterium. Bio-priming of rice seeds with BS and rutin not only augmented root and shoot lengths but also the photosynthetic pigments like chlorophyll and carotenoid. Similarly, high accumulation of phenolic and flavonoid contents was observed in the leaves. Fluorescent microscopic images revealed that BS plus rutin enhanced callose deposition in the leaves. It was also established that the least formation of reactive oxygen species in BS plus rutin treated rice plants was due to higher free radicals scavenging activity and total antioxidant potential. The results highlight chemo attractant nature of BS towards rutin, which by enhancing biofilm formation and root colonization indirectly strengthened the plants’ defensive state.

## Introduction

Dwindling soil fertility and crop productivity is the foremost global concern in order to achieve food security for the snowballing world population which is expected to reach 9.3 billion by 2050 [1]. Attaining food security for a still-enlarging global population is a large and complex challenge. In a recently conducted study it was reported that boosting soil health alone can increase productivity by 10–15% and in amalgamation with effective plant attributes, the agricultural productivity can be increased up to 50–60% [2]. In context with the above report, plant-associated microorganisms have been looked upon as potential partners to help attain the formidable goal.

Microbes and plants are well known as faithful comrades in beneficial interactions because of its important role in nutrient mobilization and uptake which is manifested by status of soil health and richness of nutrient pool. Microbes support plant health via increasing the availability of nutrients, hormonal stimulation thereby making plants more resistant to biotic and abiotic stresses during various ecological changes. However, the support rendered is not unidirectional as plants in turn provide number of organic acids, flavonoids, and carbohydrates etc. which enhance the growth and colonization potential of microbes in its vicinity [3]. The specific kind of bio molecule exudation by the plant will depend on the plant, microbes involved, and kind of stress. Recent research indicated that, some phenolic compounds such as cinnamic, ferulic and ellagic acids were found to enhance the plant forbearance to abiotic stresses like chilling, salinity and osmotic stress [4, 5, 6]. Few researches experimentally showed the role of flavonoids on stimulation of hyphal growth during early interactions between roots and mycorrhizal fungi [7, 8].However, more information should be generated towards understanding the nonsymbiotic-plant microbe interactions, as little progress has been made in identifying the molecules responsible for attracting such rhizospheric microbes towards plants.

Amongst the group of various plant growth promoting microbes, *Bacillus subtilis* is commonly found in association with roots of diversified plants [9, 10].The direct beneficial multiferious effects of *B*. *subtilis* strains include induction of induced systemic resistance, plant growth promotion and disease suppression [11]. However, the effectiveness and performance in the field is challenged and there exists a discrepancy between the desired and observed results because of the inefficient colonization around the rhizospheric zone of plants. Effective rhizosphere colonization is an essential factor not only as the first footstep in pathogenesis of soilborne microorganisms, but is also a decisive aspect in the application of microorganisms for harnessing the beneficial purposes In this perspective, secondary metabolites especially flavonoids are well known to play a crucial role in regulating numerous interactions between plants and microbes like the association between legumes and *Rhizobium* [12], plants and *Agrobacterium* [13], or early interactions between roots and endomycorrhizal fungi [7]. Keeping these in mind, the study was designed with rutin, a bioflavonoid to assess its effect on biofilm forming potential of *B*. *subtilis* CIM (BS) along with the outcome on growth, ROS-scavenging molecules, primary pigments, total phenolic and flavonoid content and callose deposition in rice seedlings. In this paper we have shown the strain *B*. *subtilis* CIM was chemotactically attracted maximally towards the picomolar concentration of rutin and plants primed with the said concentration of rutin not only behaved better because of efficient colonization by *B*. *subtilis* CIM in comparison to control plants but were also better equipped with the defence metabolites.

## Materials and Methods

### Culture and culture conditions

The *B*. *subtilis* CIM (NAIMCC-B-01816) used in the study has been selected on the basis of its previous report as plant growth promoter and biocontrol agent [14]. The culture has been deposited at the National Agriculturally Important Microbial Culture Collection (NAIMCC), Mau, India. For culturing *B*. *subtilis* CIM, it was inoculated in the Luria Bertani (LB) broth (Himedia, Mumbai, India) and kept under shaking conditions (120 rpm) at 28°C. For seed treatment, the culture obtained after 24 h was centrifuged at 6000 × *g* for 5 min and the cell density was adjusted to 10^8^ CFU mL^−1^ in saline (0.85% NaCl) using a spectrophotometer (Spectra Max, Molecular Devices) at 610 nm.

### Chemotaxis assay

Chemotaxis was studied in soft-agar swarm plates. *B*. *subtilis* CIM (OD_600_ = 0.6) was point inoculated onto the centre of 0.4% agar plates. To the left side of the *B*. *subtilis* CIM inoculum, water as control was inoculated on whatman filter paper disc while to the right side different concentrations of rutin were added to test its potentiality as chemo attractant. Soft-agar plates were incubated at 30°C for 18 to 24 h and visualised for the chemotactic movement of the bacteria.

### Motility assay

The swarming, swimming, and twitching motilities of *B*. *subtilis* CIM were investigated using the following media: (1) swim plate [1% tryptone, 0.5% NaCl, 0.3% Agar (Himedia, Mumbai, India)], (2) swarm plate (0.8% nutrient broth), 0.5% glucose, 0.5% agar), and (3) twitch plate (1% tryptone, 0.5% yeast extract, 0.5% NaCl, 1% agar). The agar media plates were air-dried for 5–10 min before use. For conducting the swimming and swarming assays, the overnight grown bacterial cells were gently inoculated using a toothpick in the centre of the solidified agar plates supplemented with 1μM, 1nM, and 1pM rutin, followed by incubation at 30°C for 20–24 h. In case of twitching motility assay, the cells were pricked into bottom of the solidified agar medium having different rutin concentrations using a toothpick and incubated at 37°C for 20 h. The colony movement on the interface between the agar medium and the dish was observed. An equal amount of only *B*. *subtilis* CIM inoculum without rutin addition in the respective media served as a control.

### Assessment of different rutin concentrations on colony forming units (CFU) of *B*. *subtilis* CIM

A known concentration of rutin (1μM, 1nM, and 1pM) was prepared in deionized water. One microlitre culture was added to the LB broth containing known concentrations of rutin. Culture was allowed to grow for 10h at 28±2°C followed by recording absorbance at 600 nm.

### Biofilm assay

#### Quantification method

Biofilm formation associated with solid surface was estimated by the crystal violet (CV) staining method, following the method of [15] with slight modifications. Briefly, an overnight grown culture of *B*. *subtilis* CIM was diluted to an OD_600_ of 0.6 and 1 μL culture was added to 99 μL LB broth in a 96-well plastic titre plate. For assessing the effect of different rutin concentrations (1μM, 1nM, and 1pM) on biofilm formation, the concentrations were maintained in the 99 μL nutrient broth filled in the titre plate. The plate was allowed to stand at 37°C for 8 h. After the specified time the surface pellicles and the cultures were carefully removed from the wells with the help of pipette. Distilled water was used to gently rinse each well and the remaining adhering cells and matrices were stained with 150 μL of 1% crystal violet (CV) solution for 25 min at room temperature. After again washing the wells twice with distilled water, the CV attached to the biofilm was solubilized in 150 μL DMSO. Quantification was done by measuring the absorbance at 595 nm.

### Microscopic visualization

Since, 1pM rutin was most effective in the above method, the effect of 1pM rutin in comparison to alone *B*. *subtilis* CIM on biofilm development was determined using polyvinyl chloride (PVC) biofilm formation assay for microscopic visualization [16]. Briefly, culture of *B*. *subtilis* CIM having OD 0.6 at A_600_ nm was added into 1 mL of LB broth medium containing 1 pM rutin for 12 h at 30°C. After the mentioned time, the wells were rinsed twice with sterile distilled water to remove the floating planktonic cells. The surface-adhered cells were stained with 0.4% CV and 0.2% methylene blue (MB) solutions, respectively. After incubation of 3 min at room temperature excess solution was removed and plates were kept for drying. The dried plates were later visualized under a light microscope at a magnification of 20X (Olympus BH2, Japan). Inhibition of biofilm formation was further examined by fluorescent microscope. Briefly, culture of *B*. *subtilis* CIM was seeded on the cover glasses under LB medium submerged conditions in the absence and presence of 1 pM rutin. After 12 h incubation period, the biofilms formed on the cover glasses were stained with 20 mM SYTO-9 green fluorescent dye and examined under fluorescent microscope (Leica DMR epifluorescence microscope, Germany).

### Experimental design

Surface sterilized *Oryza sativa* (Rice) seeds treated with pure culture of *B*. *subtilis* CIM and 1 pM rutin were raised in earthen pots (15 cm diameter) containing sterilized soil in a greenhouse under natural light conditions. The potting mixture used in the experiment consisted of autoclaved soil: compost (3:1) sieved through 2.5 mm mesh and then autoclaved for 3 h in autoclavable plastic bags. The experimental soil was sandy loam with pH 7.4, EC 0.38 dS m^−1^, 155 kg ha^−1^available nitrogen (alkaline permanganate extractable), 9.5 kg ha^−1^ available P (0.42 M NaHCO_3_ extractable), and 86 kg ha^−1^potassium (neutral N C_2_H_3_O_2_NH_4_ extractable). Following four treatments were used in the experiment: (a) Healthy control plants (b) *B*. *subtilis* CIM inoculated plants (BS) (c) 1pM rutin (Ru) (d) BS+ Ru. After 4 weeks of seed germination, the data for different physiological parameters was recorded. All biochemical parameters were assayed in the fresh leaves immediately after plucking the leaves.

### Plant growth promoting traits and colony forming units

For recording growth parameters, three random samples were uprooted from each pot after 30 days of seed germination. The plants were carefully washed under running tap water to remove the soil particles clinging to the roots. Blotting paper was used for drying the washed plants. Shoot length and root length were measured using a ruler. The density of *B*. *subtilis* CIM inoculated in presence and absence of 1 pM rutin in the soil was assessed in terms of CFU g^-1^. The agar plate count method was used to estimate the population of *B*. *subtilis* CIM using LB media.Data from the replicated pots were then pooled for analysis.

### Phytochemical analysis

The chlorophyll and carotenoid content of leaf tissue was determined according to the method of Arnon [17] and Hartmut [18]. Briefly, 100 mg of leaf tissue was powdered in mortar and pestle followed by extraction with CH_3_OH:H_2_O (9:1, v/v) mixture. The mixture was centrifuged at 8,000× g for 5 min at room temperature and absorbance of the filtrate was recorded at 663, 645, 480 and 510 nm.

The total phenolic content (TPC) was quantified using Folin–Ciocalteu method [19], based on the oxidation-reduction reaction. A 0.5 mL of FolinCiocalteu reagent and 2.5 mL of sodium carbonate solution (7%) were added to 1 mL methanolic extract of leaf tissue. The mixture was allowed to stand for 2 h at room temperature in dark and absorbance was recorded for the blue coloured end product developed at 765 nm. The results were expressed as mg gallic acid equivalent per g of dried sample.

Total flavonoid content (TFC) was analysed according to the method described by Zhishen et al. [20] using rutin (mg g^-1^) as standard. A 0.15 mL of 15% sodium nitrite solution was added to 0.5 mL of methanolic plant extract. After 6 min, 2 mL of 4% NaOH was added to make the reaction volume 5 mL with water. Absorbance at 510 nm was recorded after 15 min.

The hydrogen donating or free radical scavenging ability (FRSA) of the methanolic sample prepared from the leaves of rice was evaluated by using the stable radical DPPH (2, 2-diphenyl-1-picrylhydrazyl), which is reduced in presence of antioxidant active substances [21]. The homogenized 1 g leaf tissue from different treatments was extracted with CH_3_OH:H_2_O (1:1) mixture. Reaction was initiated by the addition of 100 μL of the extracted sample (supernatant) to 400 μL Tris-HCl (0.1M, pH 7.4) and 500 μL of freshly prepared methanolic DPPH solution (0.5 mM). The mixture was kept at room temperature for 15 min in dark conditions and absorbance was observed at 517 nm. The scavenging activity was estimated according to the following equation:

$$\text{Scavenging potential}\;\left(\%\right)=\left[\left({\text{absorbance}}_{\text{control}}-{\text{absorbance}}_{\text{sample}}\right)\;{\left({\text{asbsorbance}}_{\text{control}}\right)}^{-1}\right]\;\times 100$$

The total antioxidant capacity (TAC) was determined by phosphomolybdate method using ascorbic acid as a standard [22]. Reaction was initiated by the addition of 100 μL of methanolic leaf tissue to 1 mL of reagent solution [3.3 mL H_2_SO_4_, 335 mg Na_3_PO_4_ and 78.416 mg (NH4)_2_MoO_4_ in 100 mL]. The reaction mixture was incubated in boiling water bath for 1 h after which the absorbance was recorded at 695 nm. The antioxidant capacity was estimated as:

$$\text{Antioxidant effect}\;\left(\%\right)=\left[\left({\text{absorbance}}_{\text{sample}}-{\text{absorbance}}_{\text{control}}\right)\;{\left({\text{absorbance}}_{\text{control}}\right)}^{-1}\right]\;\times 100.$$

All *in vitro* data presented are average of three independent replicates and mean values were calculated.

### Histochemical analysis of reactive oxygen species and plant cell wall strengthening

The reactive oxygen species (ROS) produced in the leaves was detected using the fluorescent probe 5-(and 6)-carboxy-2′, 7′-dichloro dihydrofluorescein diacetate (DCF-DA) (Sigma-Aldrich, United Kingdom). Treated and untreated leaves were infiltrated with 60 μM of DCF-DA for 3 min in a standard buffer solution (1 mMKCl, 1 mM MgCl_2_, 1 mM CaCl_2_, 5 mM 2-morpholinoethanesulfonic acid (pH 6.1). Leaves were then rapidly rinsed in DCF-DA buffer and observed under a Leica DMR epifluorescence microscope with a GFP filter set (excitation 480/40 nm, emission 527/30 nm) (Leica, Germany) [23].

Plant cell wall strengthening was also histochemically analysed using 0.01% aniline blue. For callose visualization, leaf samples were cleared in boiling ethanol and examined for the presence of cell wall deposits. Detection of callose was performed using a UV epifluorescence microscope according to the aniline blue staining technique [24].

### Statistical analysis

The experimental data was expressed as the mean of three independent replications. For statistical analysis of the data, Analysis of variance (ANOVA) techniques was performed by SPSS package (SPSS V16.0, SPSS Inc., Chicago, IL) and means were separated using Tukey’s multiple comparison test at *P* < 0.05. Linear correlation coefficient was evaluated among biochemical and antioxidant parameters. The principal component analysis (PCA) was applied to produce components suitable to be used as response variables in present analysis.

## Results

### Effect of different concentration of rutin on swimming, swarming and twitching activities of *B*. *subtilis* CIM

**Fig 1:**
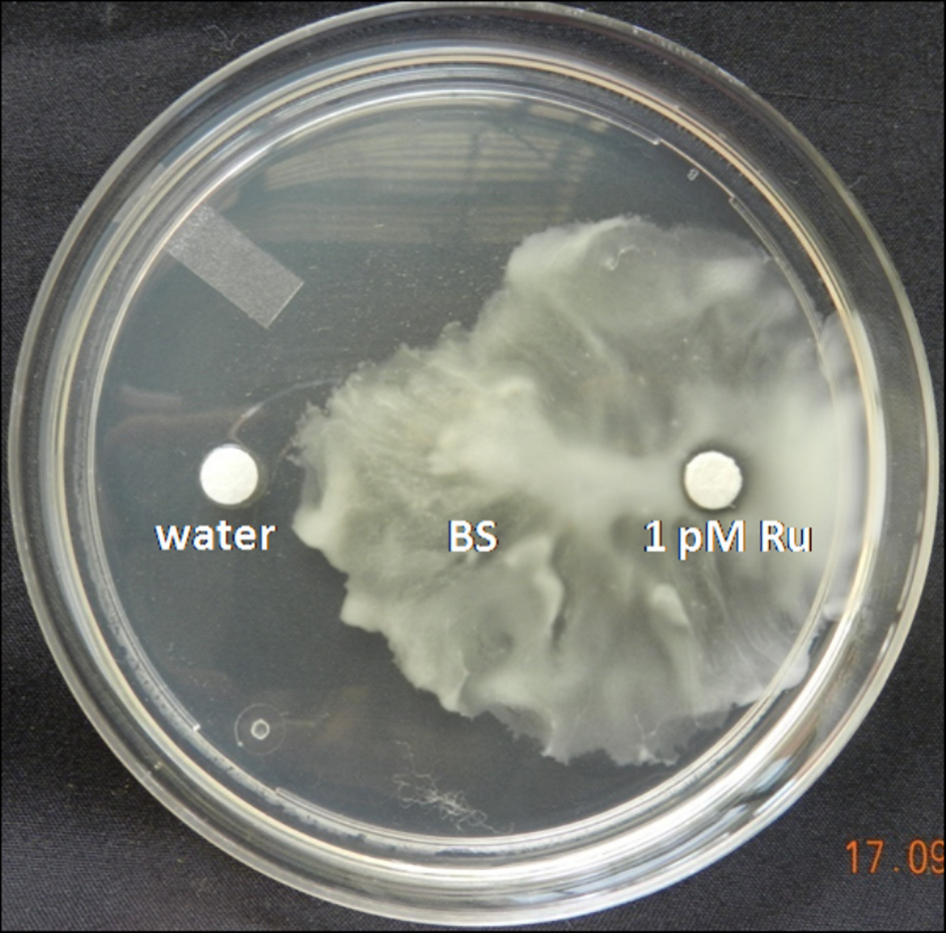
Agar plate assay showing chemotactic response of *B*. *subtilis* CIM towards 1 pM rutin. The filter paper having attractant was put on the right, water was put on the left and bacterium was spot inoculated in the middle.

**Fig 2:**
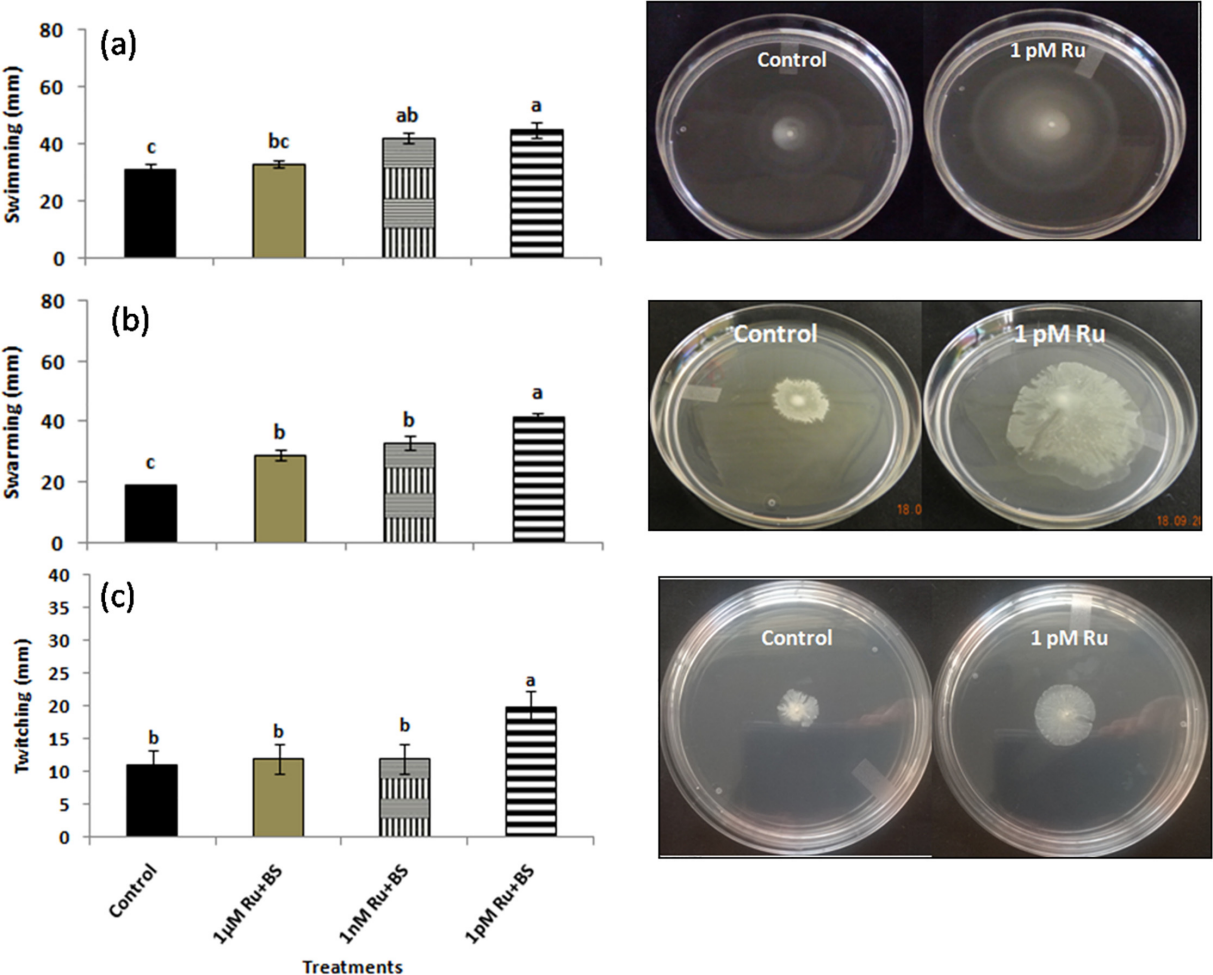
Effect of rutin on swimming (a), swarming (b), and twitching (c) of *B*. *subtilis* CIM. Cells were inoculated at the center of the agar media with or without rutin and incubated at 30°C for 20–24h.

The increased growth of *B*. *subtilis* CIM towards different concentration of rutin suggested their motile nature. Maximum dispersion of cells from the point of inoculation was observed in 1 pM rutin inoculation in comparison with other concentrations (Fig 1; S1 Fig). On varying concentrations of soft agar, *B*. *subtilis* CIM was found to be motile towards the different concentrations of rutin. When the *B*. *sublitis* CIM with different concentrations of rutin was cultivated on 0.3% soft agar plates, the zone of swimming was in the order 1 μM (1.06 fold) and 1 nM (1.35 fold) followed by 1 pM (1.45 fold) in comparison to control (Fig 2A; Figure A in S1 File). On swarm agar plates, *B*. *sublitis* CIM with different rutin concentrations showed almost similar swarm phenotype with zone of migration in the order: 1 μM (1.53 fold), 1 nM (1.74 fold) followed by 1 pM (2.21 fold) with respect to control plate (Fig 2B; Figure B in S1 File). Finally, when the strains were stabbed through a thin agar layer, the *B*. *subtilis* CIM with 1 pM rutin (1.81 fold) formed a maximum and denser zone of twitching motility at the agar interface than the 1 μM (1.09 fold) and 1 nM (1.09 fold) treatments (Fig 2C; Figure C in S1 File). A positive and strong correlation was observed among swimming and swarming (r = 0.919*); and twitching with swimming and swarming (r = 0.757 and r = 0.848, respectively) (S1 Table).

### Effect of different concentration of rutin on growth and biofilm formation of *B*. *subtilis* CIM

**Fig 3:**
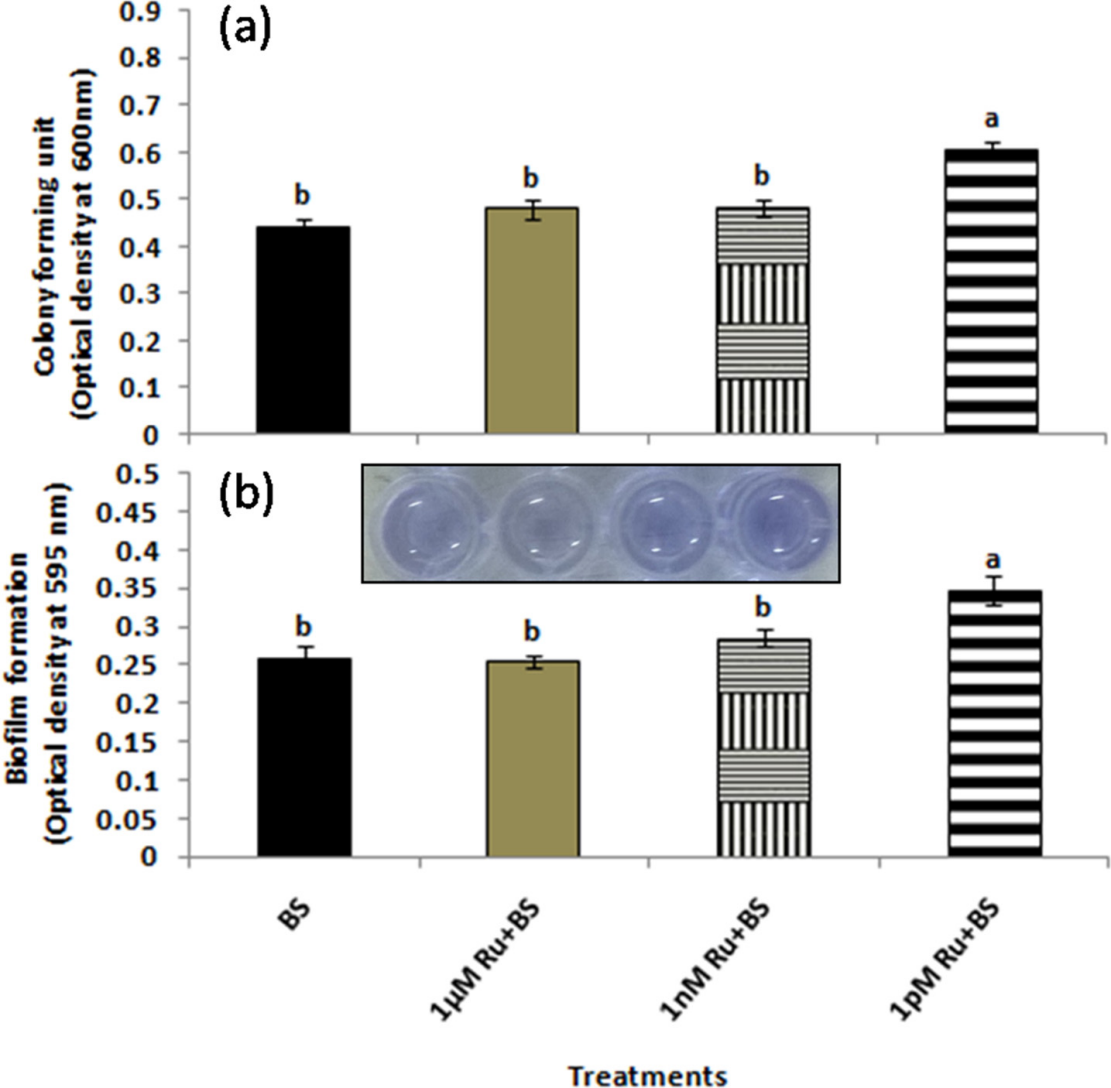
Effect of rutin on (a) colony forming units (CFU) and (b) biofilm quantification of *B*. *subtilis* CIM. Biofilm quantification was determined by crystal violet staining.

**Fig 4:**
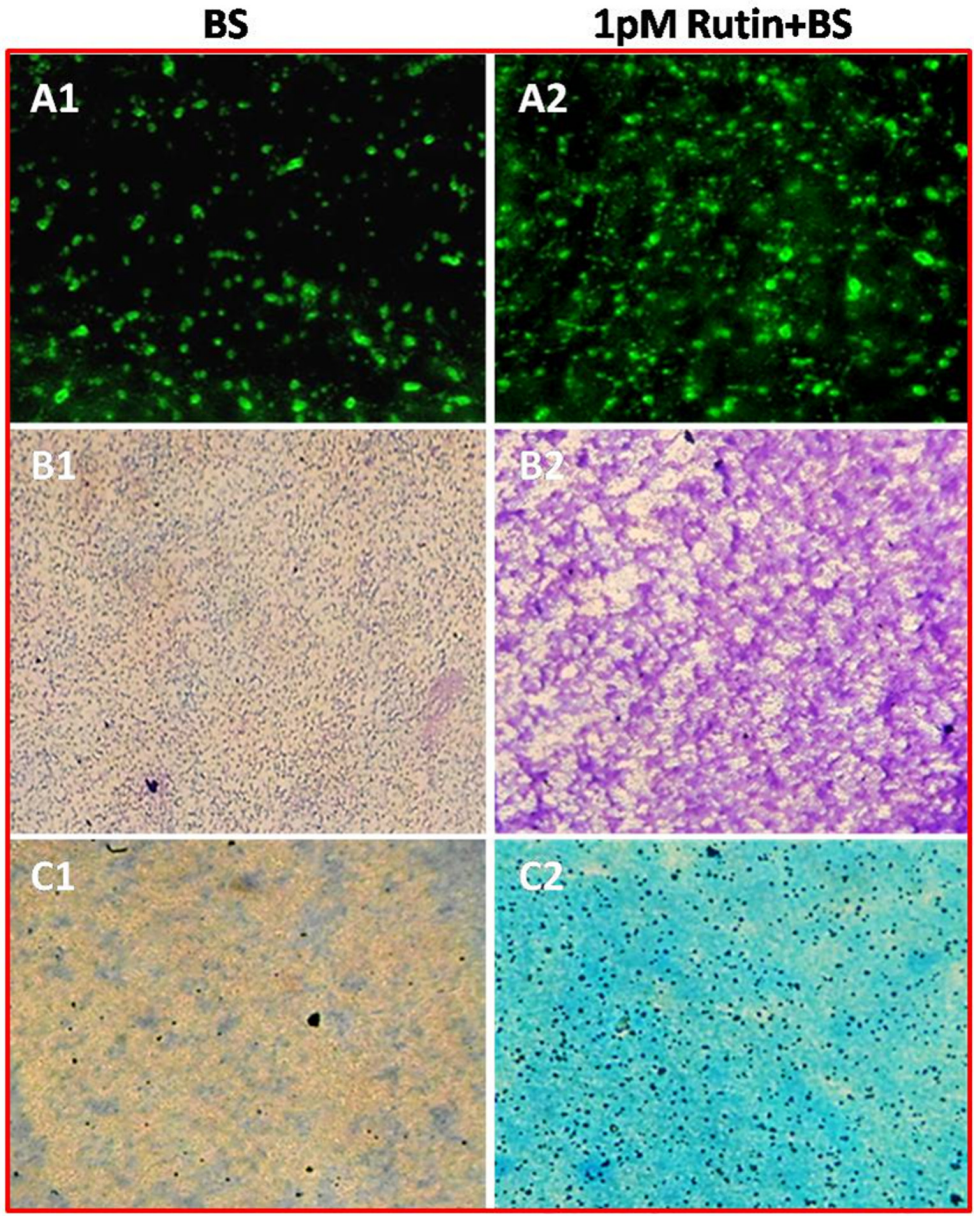
Morphology of biofilm formed examined by light microscopy. Bacterial biofilms were grown in the absence or presence of 1 pM rutin. (A1-A2) Fluorescent microscope images; (B1-B2) CV-stained light microscope images; (C1-C2) MB-stained light microscope. (20X, scale bar = 200 μm).

The growth of *B*. *subtilis* CIM in terms of CFU in LB broth containing different concentration of rutin, was significantly (*P* ≤ 0.05) higher in 1pM (1.38 fold) than the other concentrations selected (Fig 3A). In the microtiter plate assay, *B*. *subtilis* CIM with 1 pM of rutin showed significantly (*P* ≤ 0.05) greater (1.35 fold) biofilm formation potential than the other concentrations and control (Fig 3B). Direct visualization of biofilm formation by light microscopy could provide valuable information on the colonization potential of the bioagent. Results obtained from SYTO-9, CV and MB staining techniques revealed dark staining in *B*. *subtilis* CIM treated cells with 1 pM rutin due to thick coating of biofilm than the alone *B*. *subtilis* CIM treatment (Fig 4). A positive and strong correlation was observed between colony forming unit and biofilm formation (r = 0.95*). Further, biolim formation was also found to be strongly and linearly correlated with swimming, swarming and twitching activities (r = 0.889, r = 0.869 and r = 0.955 respectively) in the present investigation (S1 Table).

### Effect of *B*. *subtilis* CIM with 1 pM of rutin treatment on growth and phytochemical activities of rice

**Fig 5:**
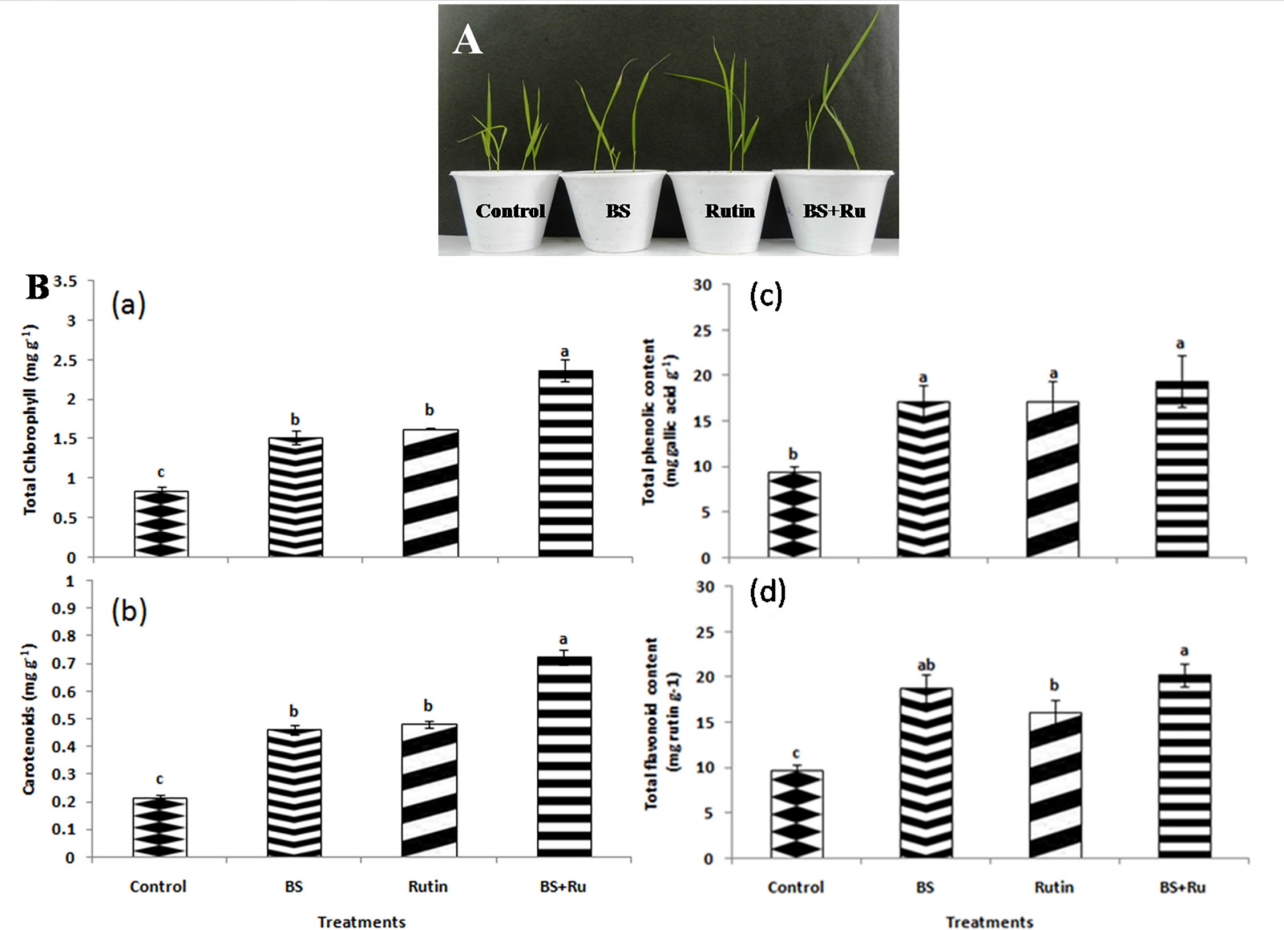
Comparison of growth and biochemical parameters between different treatments. (A) Effect of different treatments on plant growth promoting traits of rice. (B) (a) Total chlorophyll, (b) carotenoid content, (c) total phenolic and (d) total flavonoid content of rice after treatment with rutin (Ru), *B*. *subtilis* (BS), Ru+BS and control.

The glasshouse experiment showed that inoculation of rice seeds with the different treatments resulted in significant (*P* ≤ 0.05) increase in plant growth with respect to the control plants (Fig 5A). The shoot and root lengths were significantly (*P* ≤ 0.05) enhanced by the combined application of *B*. *subtilis* CIM with rutin (Table 1). The maximum increase in shoot and root length was found to be 1.31 and 1.45 folds, respectively higher in *B*. *subtilis* CIM with 1 pM rutin treatment followed by alone *B*. *subtilis* CIM and rutin treatment with respect to the control plants (Table 1). Root colonization data further supported the plant growth promotion results as maximum CFU of *B*. *subtilis* CIM was observed in picomolar rutin primed seedlings than the treatment having only *B*. *subtilis* CIM (Table 1).

**Table 1:** Effect of rutin (Ru), *B*. *subtilis* CIM (BS), BS+Ru and control on growth parameters of rice.

Treatments	Shoot length (cm)	Root length (cm)	Population density of BS (CFU×10^4^ g^-1^ of soil)
Control	14.07 ± 0.43^**b**^	2.40 ± 0.20^**b**^	-
BS	16.0 ± 0.90^**b**^	2.80 ± 0.30^**ab**^	2.83 ± 0.44
Rutin	16.17 ± 1.33^**ab**^	2.60 ± 0.10^**b**^	-
BS+Ru	18.5 ± 0.70^**a**^	3.50 ± 0.50^**a**^	3.67 ± 0.33

Results are means of three replicates ± SD and means within a column followed by the same letter are not significantly different (*P* ≤ 0.05).

**Fig 6:**
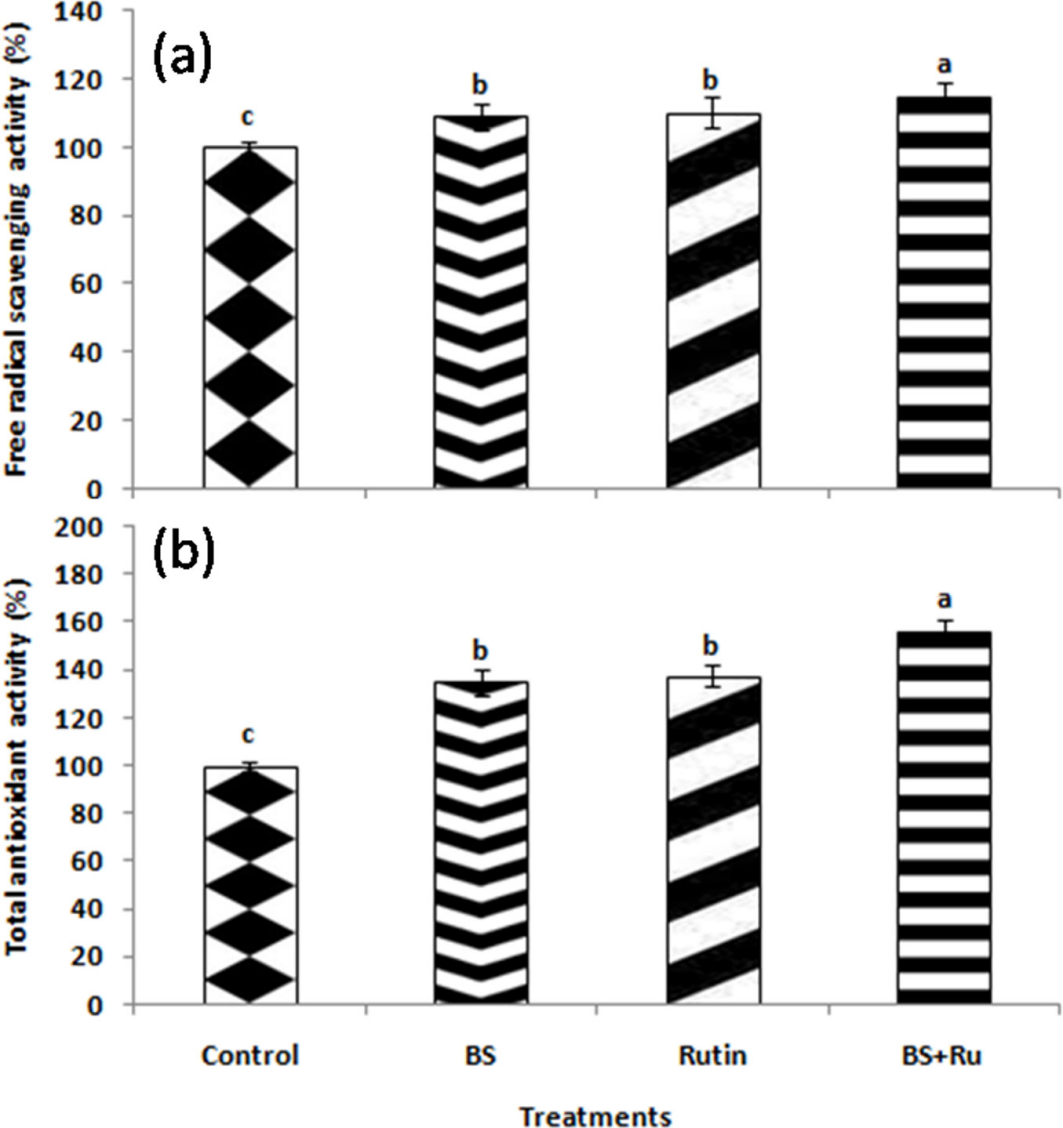
(a) Free radical scavenging activity and (b) total antioxidant capacity of rice. Different treatments used in the study included only rutin (Ru), only *B*. *subtilis* (BS), combined application of BS+Ru and healthy control.

Total chlorophyll content was increased significantly (*P* ≤ 0.05) in all the treatments (Fig 5Ba). The maximum increase in total chlorophyll and carotenoids by 2.80 and 3.34 folds, respectively was observed in BS+Ru treatment followed by BS (1.78 and 2.12 folds, respectively) and rutin (1.91and 2.22 folds, respectively) as compared to control (Fig 5Bb). Similarly, higher TPC was recorded in BS+Ru (19.37 mg gallic g^-1^) followed by BS (17.22 mg gallic g^-1^) and rutin (17.13 mg gallic g^-1^) treatments, which were about 2.05, 1.82 and 1.81folds, respectively higher than the control (Fig 5Bc). The highest TFC content was also recorded in BS+Ru (20.24 mg rutin g^-1^) followed by BS (18.74 mg rutin g^-1^) and Ru (16.12 mg rutin g^-1^), which was 2.09, 1.93 and 1.66 folds, respectively higher than the control plants (Fig 5Bd). The combination of BS+Ru significantly (*P* ≤ 0.05) increased FRSA (1.15 fold) followed by BS and rutin treatments as compared to control (Fig 6A). Similar to FRSA, maximum value of TAC was also observed in BS+Ru treatment, which was 1.56 fold higher as compared to the control (Fig 6B).

### Effect of *B*. *subtilis* CIM with 1 pM rutin treatment on ROS and callose deposition in rice leaves

**Fig 7:**
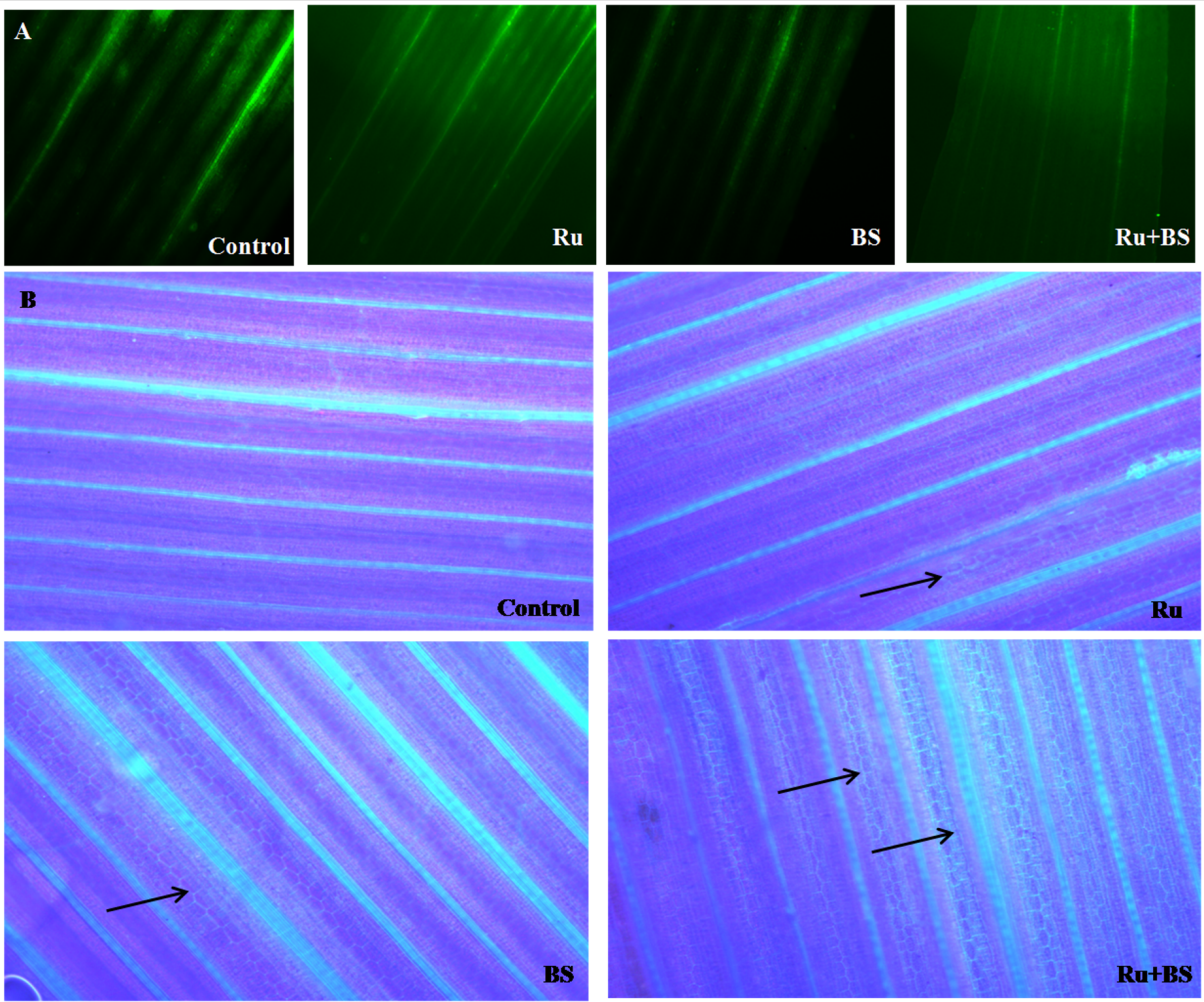
ROS production (10X, scale bar = 100 μm) (A) and callose deposition in leaves (20X, scale bar = 200 μm). (B) of rice. Different treatments used in the study included only rutin (Ru), only *B*. *subtilis* (BS), combined application of Ru+BS and healthy control. Black arrows denote callose deposition in different treatments.

Histochemical analysis of DCF-DA for ROS in rice leaves revealed higher amount of ROS localization in the mid rib region of control plants as compared to the treated ones (Fig 7A). Compared to the rutin and BS alone, least ROS localization was observed in BS+Ru treatment (Fig 7A). Interestingly, callose deposition, visualized by an intense blue-green fluorescence under UV light after staining with aniline blue, was also found to be preferentially deposited in the interveinal regions cells of rice leaves (Fig 7B). Overall, the treatment of BS+Ru, alone BS and rutin led to an increase in callose deposition in rice leaves, with most significant increase observed in BS+Ru treatment, indicating the synergistic role of BS and rutin in strengthening the defence response in plants.

### Principal component analysis

**Fig 8:**
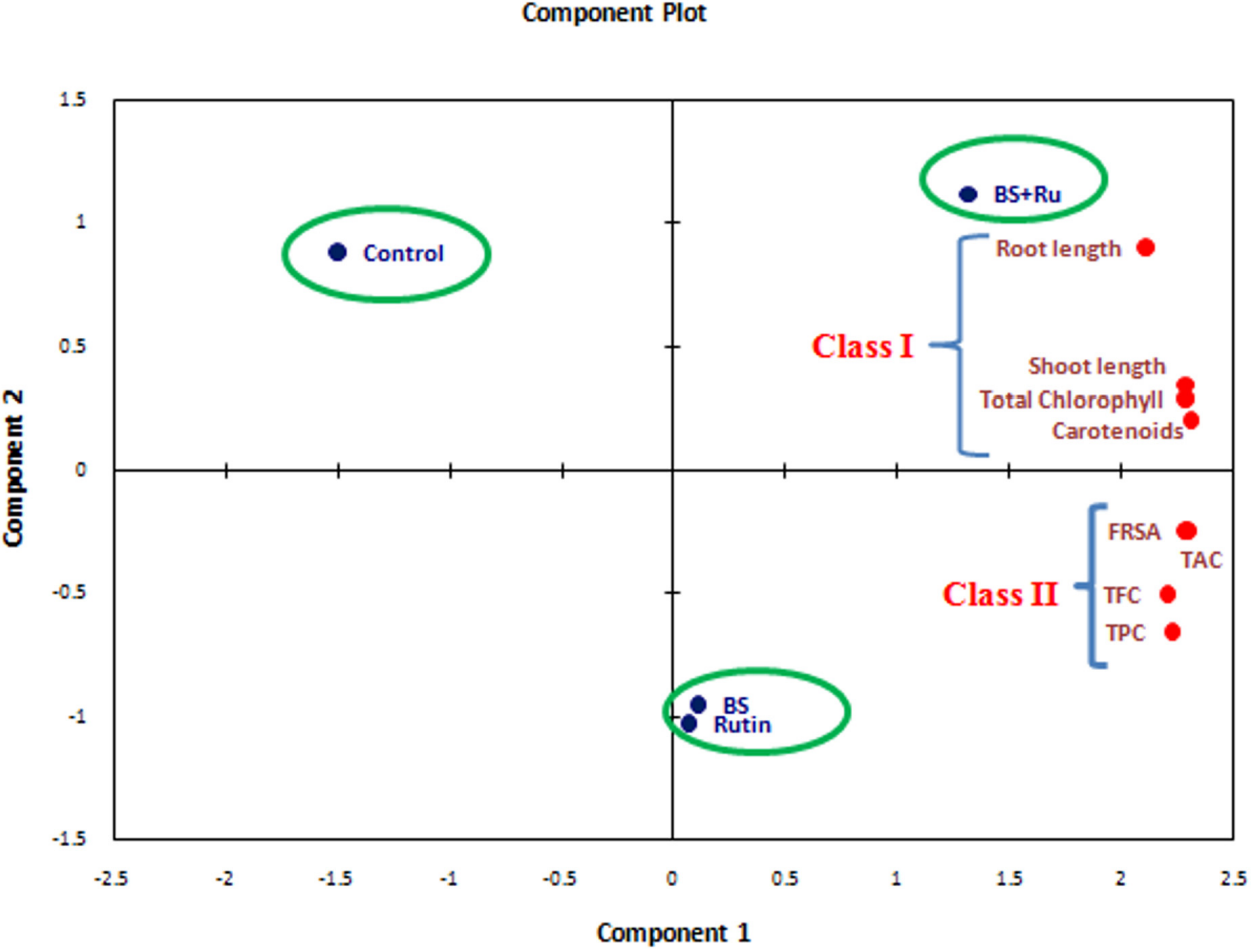
Principal component analysis (PCA) of various physiological and biochemical parameters analyzed in rice leaves.

The results obtained from the present study was further authenticated by PCA as clustering of treatments revealed formation of three different groups: one having only control, second having both BS and rutin alone treatments and third having BS+Ru. The BS+Ru treatment formed a separate group where all the growth and physiological parameters of plant were increased significantly (*P* ≤ 0.05). The PCA contributes 98.78% of the total variance where PC1 contributed 94.46%, and PC2 contributed 4.32% of the total variance (Fig 8).

## Discussion

For initiating a mutualistic association, it is prerequisite for both host plants and microbes to respond to reciprocal signals and figure out their responses for the development of a lifestyle that provides mutual benefits [25]. Here we have shown that rutin at picomolar concentration chemotactically attracted rhizospheric bacterial isolate, *B*. *subtilis* CIM,which in turn improved the growth and defence parameters in rice seedlings than the alone treatments. Recently, working on the effect of phenolic acids on plants, some groups have identified the potentiality of phenolic acids and flavonoids as seed biopriming agents for improving the plant health under normal and stress conditions [26, 27].

Bacteria use a range of motility machinery to colonize environments which includes flagellar movement dependent swimming and swarming, and flagella-independent, twitching, gliding, and sliding [28]. Interestingly, better swimming, swarming, and twitching movement on agar surface at picomolar concentrations of rutin might have acted as an environmental cue. The above explanation can be the reason for the enhanced biofilm formation which has been implicated to play a key role in efficient root colonisation thereby exerting beneficial effects on plant growth. Recently, a study showed that effective *B*. *subtilis* colonization and biofilm formation in *Arabidopsis thaliana* roots was triggered by certain plant polysaccharides [29]. Similarly, in another study tricarboxylic acid cycle intermediate L-malic acid (MA) secreted from roots of *A*. *thaliana* selectively signalled and recruited the beneficial rhizobacterium *B*. *subtilis* FB17 chemotactically in a dose-dependent manner [30].

Beneficial soil bacteria bestow plants with immunity against an extensive range of plant diseases by triggering plant defenses, enhancing growth parameters and secondary metabolites. Here, we showed that plant growth promoting traits namely shoot length and root length were increased in treatment having *B*. *subtilis* CIM with picomolar rutin concentration probably because of the effective root colonisation by the bacterial isolate. Furthermore, 1pM rutin was also found to stimulate significantly the inducible defense responses of rice in comparison to the control and alone treatments. Amongst the various physiological parameters, leaf chlorophyll content is an important trait directly linked with the photosynthetic efficiency of the plants. In the present study, flavonoid application enhanced the total chlorophyll and carotenoid content as reported by Zhang et al. [31] who showed augmented photosynthetic capacity in Arabidopsis by application of a plant growth promoting soil bacterium *B*. *subtilis* GB03.

Flavonoids and phenolics acids are the bioactive secondary metabolites produced in plants for their role in plant defence. The results presented herein showed that seed priming with 1 pM rutin followed by inoculation with *B*. *subtilis* CIM significantly improved the phenol and flavonoid content in rice leaves. Results obtained are in corroboration with the previous findings of Fokom et al. [32] who showed significant increase in phenol and flavonoid content of cowpea upon application of flavonoid (3-o-glucoside kaempferol). Further, rice pretreatment with rutin followed by *B*. *subtilis* CIM inoculation also resulted in increase in free radical scavenging activity and total antioxidant content which could be possibly due to the positive effect of priming in regulating the shikimic acid biosynthetic pathway as thephenolic acids produced are well known freeradical scavengers [33]. ROS production needs to be homeostatically regulated for the normal functioning of both plant and animal system. In the present investigation, enhanced free radical scavenging potential was further validated by fluorescent images of ROS localization in leaves as less ROS accumulation was observed in rutin primed and *B*. *subtilis* CIM colonized seedlings with respect to control plants. Further, rutin pre-treated seedlings had significantly higher values of TAC as compared to the unprimed counterpart probably because of the potentiality of rutin to attract more *B*. *subtilis* CIM in the rhizospheric zone which in turn induced significant accumulation of cellular antioxidants such as phenolic acids and flavonoids. In this regard, Abu El-Soud et al. [4] reported increased antioxidant capacity of chickpea seedlings subjected to osmotic stress upon ellagic acid priming. The PCA results showed that attributes such as shoot length, root length, total chlorophyll and carotenoids contents significantly influenced the other parameters of rice plants such as accumulation of total phenolic and flavonoids, free radical scavenging activity and total antioxidant capacity which in turn contributed to the overall plant growth and development.

Apart from the biochemical changes, plant cell wall alterations have been recognized as a defence response initiated by plants to protect itself from the attack of harmful pathogens [34]. In primed plants, defense responses like deposition of callose, lignin, defence enzymes, and antioxidants etc. are not triggered straightway, but are stepped up profusely under stress conditions [35]. In a study conducted by Raj et al. [36], *Bacillus pumilis* strain INR-7 significantly elevated lignin, callose, and H_2_O_2_ deposition upon *Sclerosporagraminicola* inoculation. It thus seems reasonable that colonisation of beneficial microbes play an important role as enhanced callose deposition was observed in rutin primed plants inoculated with *B*. *subtilis* CIM.

**Fig 9:**
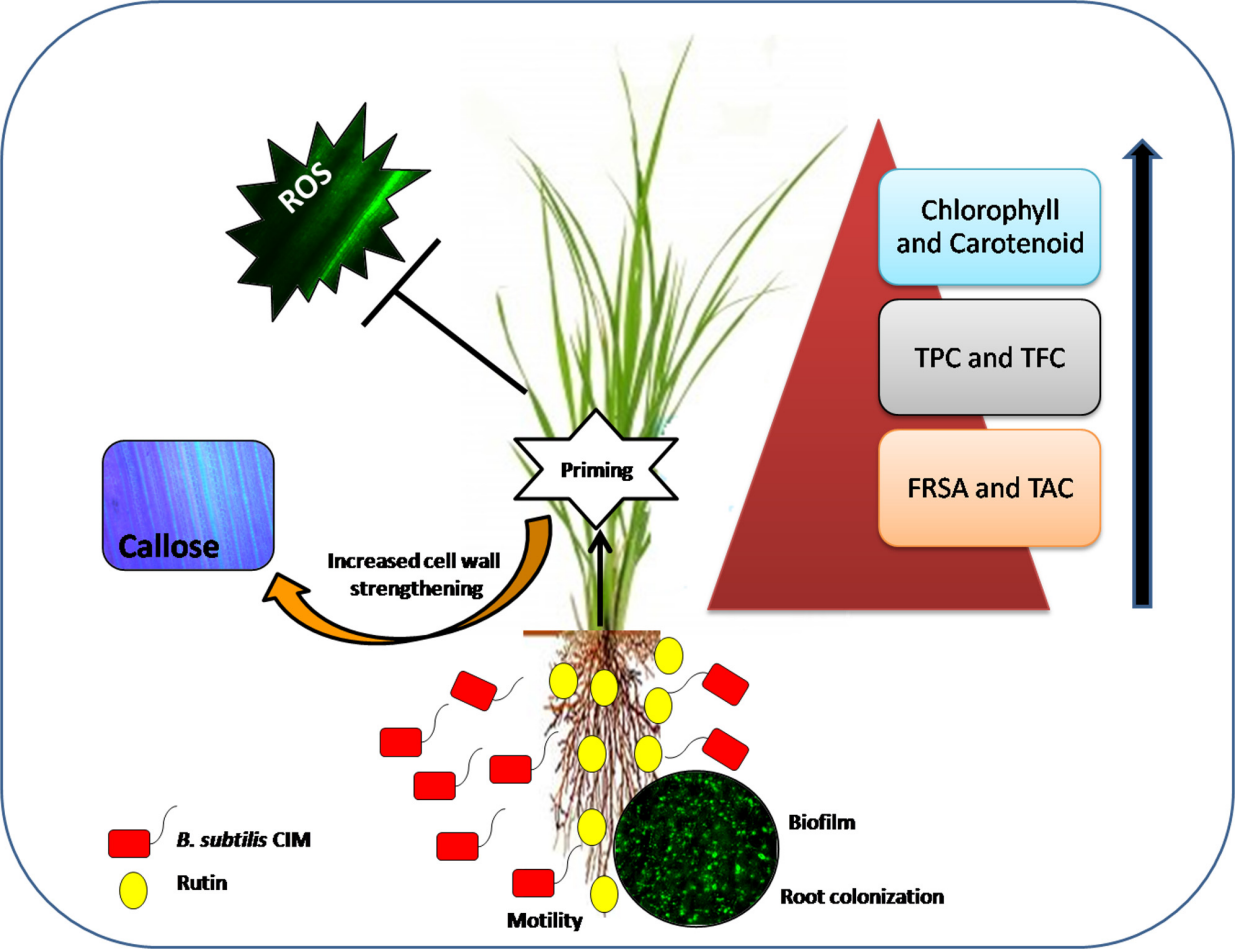
Schematic representation of the interactive mechanism of action of rutin and *B*. *subtilis* CIM.

Recently, global attention is towards the development of farming techniques, which are not only socio economically balanced but also augment crop production and protection, in an eco-friendly manner. Based on the above thought, present work sheds light on the role of a plant flavonoid, rutin in successful establishment of *B*. *subtilis* CIM in rice plants thereby providing competitive advantage above the other plants (Fig 9). This research can be further beneficial for the development of *B*. *subtilis* CIM based bioformulation with rutin as an active ingredient which will not only improve productivity but will also enhance the defensive state of plants. However, other modes of rutin action could not be ruled out and more research at molecular level will unravel the exact mode of interaction.

## Supporting Information

S1 Fig Agar plate assay showing chemotactic response of *B*. *subtilis* CIM towards 1 μM and 1 nM rutin. The filter paper having attractant was put on the right, water was put on the left and bacterium was spot inoculated in the middle.
(TIF)

S1 File Effect of rutin on swimming (Figure A), swarming (Figure B), and twitching (Figure C) of *B*. *subtilis* CIM. Cells were inoculated at the centre of the agar media with or without rutin and incubated at 30°C for 20–24h.
(TIF)

S1 Table Correlation among swimming, swarming, twitching, CFU and biofilm at different concentrations of rutin inoculated with *B*. *subtilis* CIM. (DOCX)
