# Supplementary figures and images for: Rice Seed Priming with Picomolar Rutin Enhances Rhizospheric Bacillus subtilis CIM Colonization and Plant Growth
Source: PLoS One. 2016 Jan 7;11(1):e0146013. doi: 10.1371/journal.pone.0146013 (PMC4711789; doi:10.1371/journal.pone.0146013)

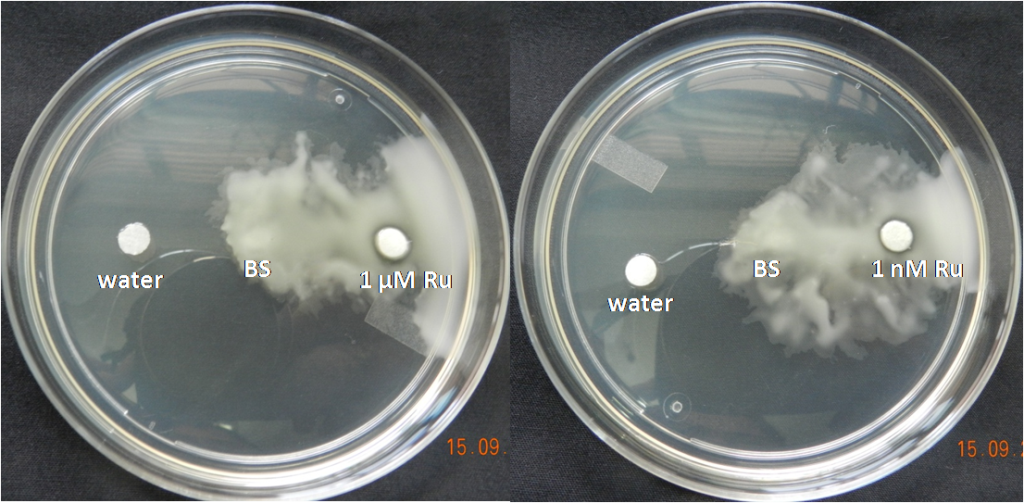

Supplement: S1 Fig — The filter paper having attractant was put on the right, water was put on the left and bacterium was spot inoculated in the middle. (TIF) [file pone.0146013.s001.tif]

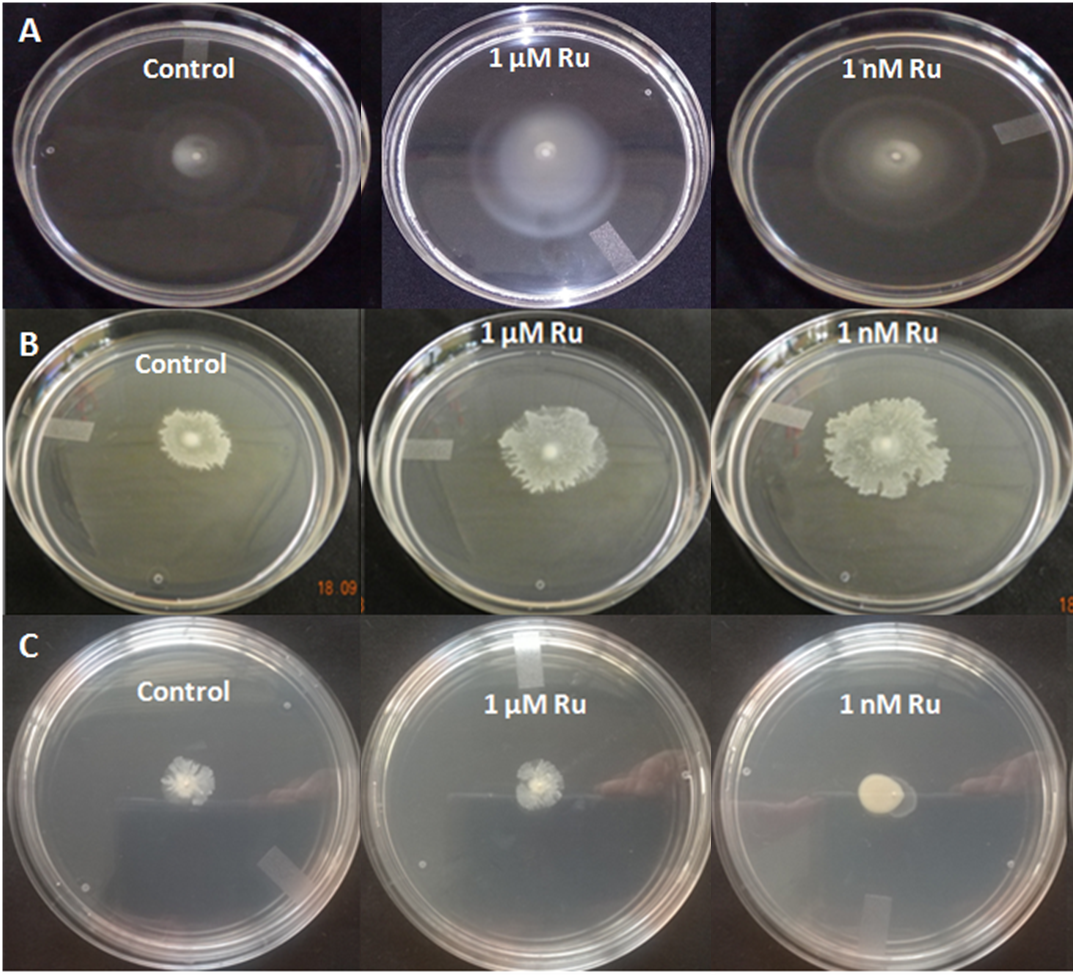

Supplement: S1 File — Cells were inoculated at the centre of the agar media with or without rutin and incubated at 30°C for 20–24h. (TIF) [file pone.0146013.s002.tif]
