# Supplementary material for: Rice Seed Priming with Picomolar Rutin Enhances Rhizospheric Bacillus subtilis CIM Colonization and Plant Growth
Source: PLoS One. 2016 Jan 7;11(1):e0146013. doi: 10.1371/journal.pone.0146013 (PMC4711789; doi:10.1371/journal.pone.0146013)
Supplement: S1 Table — (DOCX) [file pone.0146013.s003.docx]

**S1Table. Correlation among swimming, swarming*,* twitching, CFU and biofilm at different concentrations of rutin inoculated with *B. subtilis* CIM.**

| Variables | Swimming | Swarming | Twitching | CFU | Biofilm |
| --- | --- | --- | --- | --- | --- |
| Swimming | 1 | 0.919 | 0.757 | 0.814 | 0.889 |
| Swarming |  | 1 | 0.848 | 0.919 | 0.869 |
| Twitching |  |  | 1 | 0.988* | 0.955* |
| CFU |  |  |  | 1 | 0.951* |
| Bioflim |  |  |  |  | 1 |

*Correlation is significant at *P* < 0.05.
